# Neuropeptide B and Vaspin as New Biomarkers in Anorexia Nervosa

**DOI:** 10.1155/2018/9727509

**Published:** 2018-06-10

**Authors:** Teresa Grzelak, Marta Tyszkiewicz-Nwafor, Agata Dutkiewicz, Aniceta Ada Mikulska, Monika Dmitrzak-Weglarz, Agnieszka Slopien, Krystyna Czyzewska, Elzbieta Paszynska

**Affiliations:** ^1^Division of Biology of Civilization-Linked Diseases, Department of Chemistry and Clinical Biochemistry, Poznan University of Medical Sciences, Swiecickiego 6, 60-781 Poznan, Poland; ^2^Department of Child and Adolescent Psychiatry, Poznan University of Medical Sciences, Szpitalna 27/33, 60-572 Poznan, Poland; ^3^Psychiatric Genetics Unit, Department of Psychiatry, Poznan University of Medical Sciences, Rokietnicka 8, 60-806 Poznan, Poland; ^4^Department of Integrated Dentistry, Faculty of Conservative Dentistry and Endodontics, Poznan University of Medical Sciences, Bukowska 70, 60-812 Poznan, Poland

## Abstract

**Introduction:**

The aim of the study was to assess the correlation between the levels of neuropeptide B (NPB), neuropeptide W (NPW), vaspin (VAS), and the total antioxidant status (TAS) in the blood, as well as nutritional status of patients with anorexia nervosa (AN).

**Materials and Methods:**

The study covered a cohort of 76 female teenagers, including 46 females with extreme AN and 30 healthy peers (CONTR) aged 12-17.

**Results:**

AN persons were characterized by higher (in comparison to CONTR) NPB and VAS concentrations and lower values of TAS levels, body weight, and anthropometric values. Positive correlations between NPB and VAS levels were noted in the AN group (R=0.33; p<0.001) as well as between concentrations of NPW and VAS in the same group (R=0.49; p<0.001). Furthermore, positive correlations existed between NPB and NPW concentrations across the whole studied population (AN+CONTR; R=0.75; p<0.000001), AN (R=0.73; p<0.000001) and CONTR (R=0.90; p<0.0005).

**Conclusions:**

In detailed diagnostics of AN it is worth considering testing NPB and VAS levels.

## 1. Introduction

Anorexia nervosa (AN) is a disease entity that is not fully understood in respect to its aetiology. Its prevalence is on the rise and treatment effectiveness is not satisfactory; approximately 10% of patients die (the highest mortality among mental diseases) [1–3]. Normalization of body weight in affected persons usually does not mean that all disease symptoms subside as patients still exhibit a number of symptoms that include both eating and depression disorders [3, 4]. The disease affects a few times more frequently females than males, in particular girls in adolescent years when significant changes take place with regard to fat tissue distribution [1, 2, 5]. AN is characterized by changes in eating habits that lead to obtaining and maintaining body weight, which is significantly lower than that expected for a given sex and age (at least 15% below the norm), which often leads to emaciation and even death [6, 7]. Recent guidelines of the American Psychiatric Association classify progression of the disease according to the BMI scale where mild AN is ≥17.00 BMI, moderate AN is 16-16.99 BMI, severe AN is 15-15.99 BMI, and finally extreme AN is <15.00 BMI [6].

Avoiding food intake, provoking vomiting, and using laxatives or diuretic agents while increasing energy expenditure of the body, mainly through intense physical activity, are behaviours often exhibited by patients with anorexia in order to obtain/maintain a very thin appearance [4]. Many patients are put under care of specialists too late as, among other things, they hide symptoms of the disease for a long time from their social environment [3]. Quantitative and qualitative malnutrition long before diagnosis is made as well as the prolonging of such a condition despite specialist treatment which combine to form a negative prognostic factor regardless of the applied therapy [7, 8]. Malnutrition mainly leads to endocrinological and cardiological disorders, as well as to multiple abnormalities within the digestive, skeletal, and reproductive system. Changes mentioned above are accompanied by hematological and biochemical changes, among others [7, 9, 10]. In recent years a few new molecules and parameters have been described that could play a significant role in regulation of energy homeostasis. Thus it could be suggested that those newly discovered molecules could be of importance in both diagnostics and prognosis of anorexia nervosa progression; they include neuropeptide B [11, 12], neuropeptide W [13–15], and vaspin [16–18] but their specific role and involvement in the disorder are poorly understood (in the case of neuropeptide B and neuropeptide W suggestions have been based only on animal studies). Neuropeptide B and neuropeptide W are endogenous ligands of GPR7 (named also NPBW1) and GPR8 (named also NPBW2, lack of gene expression for GPR8 in rodents) receptors, which take part in energy homeostasis and hypothalamic-pituitary-adrenal axis regulation [11]. An adipokine named vaspin (visceral adipose tissue-derived serine protease inhibitor) is produced mainly in visceral adipose tissue, but also in the cells of hypothalamus and in some parts of digestive system (i.e., mucous membranes of the stomach, liver, and pancreas) [19, 20]. This may be linked with some symptoms of anorexia nervosa. Previous researches have shown increase of serum vaspin levels in anorexia nervosa compared with healthy controls [16].

Attempting to understand which certain factors influence the maintaining of pathological behaviours and attitudes towards food among patients with anorexia nervosa could contribute to improving the effectiveness of therapy and minimizing the risk of a relapse. The aim of the study was to assess the correlation between the levels of neuropeptide B, neuropeptide W, vaspin, and the total antioxidant status in the blood, as well as nutritional status of patients with anorexia nervosa.

## 2. Materials and Methods

### 2.1. Characteristics of the Studied Population

The study covered a cohort of 76 female teenagers, including 46 females with extreme AN (showing all of the symptoms of restrictive or bingeing-purging subtype of AN, including amenorrhea, with BMI<15 kg/m^2^) and 30 healthy peers (CONTR) aged 12-17. Men were excluded from the study because of small number of adolescent males suffering from anorexia nervosa and hospitalized in the Department of Child and Adolescent Psychiatry because of extreme anorexia nervosa [2].

The AN group consisted of inpatients admitted to the Department of Child and Adolescent Psychiatry. The age of onset was between 12 and 17; most of the patients were hospitalized because of eating disorders for the first time. The diagnosis of anorexia nervosa was made by two independent psychiatrists from the Department of Child and Adolescent Psychiatry according to WHO guidelines (ICD 10 [code F 50.1]) [21]. All examined patients had their anthropometric measurements taken after overnight fasting (body weight measurements and height using a certified scale with accuracy to 0.1 kg and a stadiometer (measurement accuracy: 0.1 cm) in light underwear and no shoes). The measurements were then used to calculate anthropometric indices: Body Mass Index, Body Mass Index z-score, and Body Mass Index-for-age percentile taking into account norms for sex and height of Polish women [22].

After securing blood samples for laboratory tests, the patients were first provided with proper hydration and nutrition during their stay at the hospital to reduce as much as possible the risk of fatal somatic complications. At the same time, the patients received psychological treatment and some of them also received pharmacological treatment, including antidepressants for patients exhibiting depressed mood and/or anxiolytic drugs, for patients who exhibited some symptoms of anxiety disorders, especially obsessions and/or compulsions [7, 23, 24]. Some of patients, who suffered from gastrointestinal problems, received also medications that support the digestive system.

The control group consisted of healthy females at similar age, height, and education level to participants of AN group. Exclusion criteria in the control group included abnormal body weight, health problems (mental and somatic), including eating disorders (also in the past and concerning first-level relatives), and addiction to psychoactive substances which was confirmed by physical examination and laboratory analyses following a detailed interview. Patients with other cooccurring psychiatric disorders were excluded from the group of patients with anorexia nervosa. The study was carried out in accordance with the Declaration of Helsinki and all who qualified for the analyses and their guardians had been informed about the course of the study and gave their consent in writing. The local Bioethics Committee gave its permission to conduct the research project (No. 329/15).

### 2.2. Biochemical and Statistical Analyses

Samples of venous blood (about 5 mL) were collected in the morning (7:00-8:00) from every participant after overnight fasting and an all-night rest. Most of the biochemical analyses were conducted immediately after collecting blood samples; however parts of the samples, which were needed to determine peptides and proteins, among others, were appropriately separated, secured,, and frozen in -80°C temperature. Analysis of neuropeptide B (Human NP-B ELISA kit, catalogue No. 201-12-7304, Shanghai Sunred Bio (SRB) Technology Co., Ltd, Shanghai, China, assay range 5–1000 pg/mL), neuropeptide W (Human NP-W ELISA kit, catalogue No. 201-12-5617, Shanghai Sunred Bio (SRB) Technology Co., Ltd, Shanghai, China, assay range 2.5–720 pg/mL), and vaspin concentration (Human Vaspin ELISA Kit, catalogue No. E106, Mediagnost, Reutlingen, Germany, assay range 4–4000 pg/mL) in blood serum was carried out using immunoenzymatic tests following the guidelines of manufacturers and taking into account double-checking of samples. Relative cross-reactivity with related peptides (in equal concentration as in human serum) did not exceed 0.006%. More details about validation and quality control of used test sets, manufactured in the certified laboratories, are available at the websites of manufacturers [25–27] and also described in previous publications [18] and additionally validated in our laboratory [unpublished data]. Microtiter plates with fixed primary antibody (specific for the assayed molecules) were incubated with blood serum (containing the antigen, namely, the measured peptide/protein) and later with secondary antibody marked with peroxidase. At the end a reaction was caused with a substrate for chromogene and the absorbance was read on a MR-96 microplate reader manufactured by CLINDIAG SYSTEMS B.V.B.A. (Pollare, Belgium). Values of peptides/protein concentration were calculated on the basis of a calibration curve determined using a 4-parameter algorithm (SigmaPlot 11.00 software) prepared each time for a specific set of assays. Coefficients of Variation (CVs) were, respectively, intra-assay (CV intra-assay) 3.5% for NPB and 6.1% interassay (CV interassay), whereas they were for NPW 4.3% and 6.2% and for vaspin 2.5% and 3.0%.

Total antioxidant status (TAS) in serum was assayed using a test by Randox Laboratories Ltd (Crumlin, Great Britain). Analysis of TAS level involved incubation of a patented molecule 2,2′-azino-bis(3-ethylbenzothiazoline-6-sulphonic acid) (ABTS) with peroxidase (metmyoglobin) and hydrogen peroxide, which produced an ABTS+ radical action with characteristic colour detected at 600 nm wavelength [using MR-96 microplate reader by CLINDIAG SYSTEMS B.V.B.A. (Pollare, Belgium)]. Results of TAS level were presented as an equivalent of Trolox (mmol/L). Intra-assay CV for the assayed parameter was 4.0% and interassay CV was 5.9%.

The obtained results of biochemical and anthropometric analysis were subject to statistical analysis using STATISTICA 12.5 software with Medical Set (StatSoft Inc., USA), including, among other things, analyses for comparing medians for unrelated (Welch test and Mann–Whitney U test) data, correlation between the studied variables (Spearman's and Pearson's tests), area under curve calculations (AUC), and area under ROC (Receiver Operating Characteristic) curve. Results were presented as means ± standard deviations and medians (with upper and lower quartile). Normality of the distribution of data was checked using Shapiro-Wilk test. Homogeneity of variance was tested using Levene's test and the statistically significant level of error was established at p<0.05.

## 3. Results

**Figure 1:**
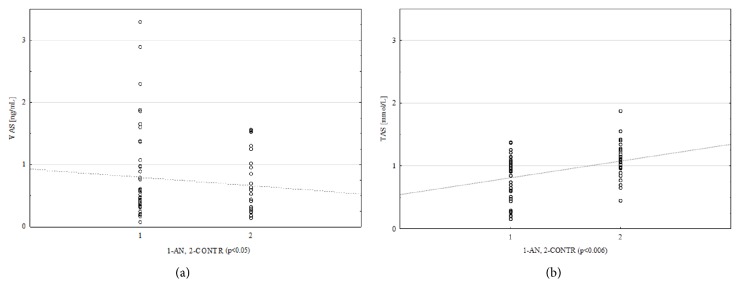
Scatter plot in the case of VAS (a) and TAS (b) values for AN (1) and CONTR (2) groups. VAS: vaspin [ng/mL]; TAS: total antioxidant status [mmol/L], AN: anorexia nervosa group, and CONTR: control group.

Persons with extreme anorexia nervosa (AN) were characterized by lower (in comparison to CONTR group) values of body weight median (by 50%; p<0.000001) and lower anthropometric values, Body Mass Index (by 45%; p<0.000001, Table 1), despite similar age and height. In AN group there were higher median concentrations of NPB in blood (by 62%, p<0.05) and VAS (by 43%, p<0.05) as well as lower medians for TAS levels (by 18%, p<0.006) compared to the CONTR group (Figure 1).

**Figure 2:**
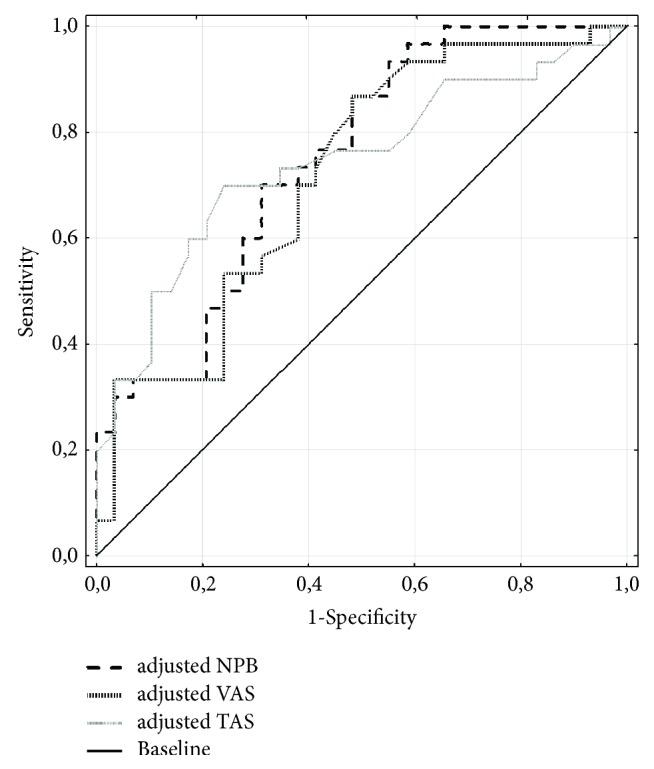
Receiver Operating Characteristic curves for adjusted serum NPB, VAS, and TAS levels according to BMI for the differentiation between AN (anorexia nervosa) and CONTR (control) groups. NPB: neuropeptide B [ng/mL]; VAS: vaspin [ng/mL]; TAS: total antioxidant status [mmol/L]; BMI: Body Mass Index [kg/m^2^].

Positive correlations between values of neuropeptide B and VAS concentrations were noted in the AN group (R=0.33; p<0.001), as well as between concentrations of NPW versus VAS in the same group (R=0.49; p<0.001). Furthermore, positive correlations existed between values of neuropeptide NPB versus NPW concentrations across the whole studied population (AN+CONTR; n=76; R=0.75; p<0.000001), AN (n=46; R=0.73; p<0.000001), and CONTR (n=30; R=0.90; p<0.0005). Comparative analysis of ROC curves (graphic representation in Table 2, Figure 2) has shown adjusted serum NPB level (AUC=0.700) is the best predictive parameter among the analyzed ones for anorexia nervosa in its extreme stage, whereas adjusted serum VAS level (AUC=0.647) proved to be a slightly less sensitive and specific measurement. Low AUC values were obtained for adjusted serum TAS level (Table 2).

## 4. Discussion

The examined patients with anorexia were characterized by elevated concentration of NPB in blood serum after overnight fasting, low body weight, and other low anthropometric values: Body Mass Index, Body Mass Index z-score, and Body Mass Index-for-age percentile compared to their healthy peers. There is no data in the literature showing the concentrations of this neuropeptide in the blood serum of healthy human subjects as well as in patients diagnosed with anorexia nervosa. Therefore, the future investigation is required. Individual analyses carried out on rodents have shown the influence of NPB on eating habits in animals but the changes depended on dosage and sex of laboratory animals. Introduction of NPB in concentrations 3 nmol/L and 10 nmol/L to cerebral ventricles in large doses decreased food intake, whereas in low doses it increased food intake for the first 2 hours and then had a proanorexic effect [11, 28].

Male NPB knockout mice were characterized by higher body weight (despite similar food intake and physical activity) compared to wild mice [NPB (+/+)] but such changes were absent when it comes to female population [12]. Adding NPB to a culture of isolated adipose cells increased lipolysis and reduced leptin secretion and expression in rat adipocytes [29]. Furthermore, adult male mice with a knockout gene for GPR7 receptor (the ligands for which are NPB and also NPW), namely, GPR7(-/-), were characterized by increased values of leptin, glucose, and insulin levels related to, among other things, hyperphagia and decreased physical activity. Such modifications are not observed in the case of female mice GPR7(-/-) [30]. There was no explanation for the sex dimorphism in terms of how NPB works in laboratory animals, as there is no explanation for greater prevalence of anorexia nervosa in human females compared to males. Disturbance in leptin levels indicates that fat tissue may play a role and as it is known that the female population (in both humans and mice) has more fat tissues than males (even in the prepubescent period) [31].

The conducted studies show that there is a positive correlation between NPB and NPW concentration in all studied groups, whereas when it comes to the control population this relationship was more pronounced in case of female patients with anorexia nervosa. It was shown that the two neuropeptides exhibit sequence homology for amino acids at a high level (66%) in case of humans [11] but their expression and activity seem to be varied. Both neuropeptides are endogenic ligands, meaning they are orphan receptors coupled with G proteins, GPR7 and GPR8 (G-protein-coupled receptors) in humans; it was shown that NPB is more effective in blocking cAMP production with the help of GPR7 receptor rather than GPR8 receptor, whereas NPW was similarly effective for both [32, 33]. In the study conducted on a group of female patients with anorexia the values of NPW concentration were higher (statistically insignificant) than in the control group. It is worth mentioning that the studied patients were of different ages (12-17) and had different anthropometric measurement results (BMI from 11 to 15 kg/m^2^) and hence probably the diversity of neuropeptide concentrations and the presence of positive correlations between NPW and VAS. Studies carried out on rodents suggest that NPW affects eating habits mainly in the male population [13] but not female, although results of the studies on the subject are inconclusive [13, 34, 35].

After introduction of NPW into cerebral ventricles using continuous infusion via an osmotic pump it was noted that in the case of animals the food intake decreased and weight gain slowed down, whereas the specific blocking of this neuropeptide (using anti-NPW IgG antibodies) reversed this effect [13]. There are also works that confirm changes in the* NPW* gene expression in the female population depending on the type of nutrient used. It was shown that intragastric administration of protein meal or glucose via a tube stimulated increase in mRNA expression for* NPW* in stomach muscles. Such interrelation was not established in case of intervention that used fat and regardless of the nutrient administered to the stomach, the intervention had no influence on NPW concentration in blood [35]. A three-month diet based on high-fat additives in female mice did not change* NPW* gene expression in the stomach, compared to using regular mice food, which would lower significantly after a few hours of overnight fasting [34]. It is worth emphasizing that dietary restrictions that concern anorexic patients include protein products, high-carbohydrate products, and especially high-fat products [8, 36].

The conducted study shows that high VAS concentrations in the group of anorexic patients suggest the influence of this adipocytokine on body weight in this disease. The significance of vaspin levels in blood in the case of anorexia nervosa was also emphasized in the work of Oswiecimska et al. from 2016 in which concentration medians of these adipocytokines were, similarly to the presented research, higher for female teenagers with anorexia nervosa at different progression stages of the disease (BMI between 11.4 and 17.3 kg/m^2^) in comparison to their peers who had a BMI level between 16.7 and 24.6 kg/m^2^ [16]. Additionally, we noted a high concentration of vaspin in the saliva of female teenagers with anorexia nervosa when compared to a group of persons with normal weight [18].

Studies on rodents have shown the many times stronger anorexigenic effect of vaspin in comparison to leptin, which consisted in limiting food intake and lowering of body weight after this adipocytokine, was administered to cerebral ventricles in daylight phase or intraperitoneally where it would remain up to 24 h after a single dose. The analyses have shown that the way this adipocytokine works is related to the Stat3 signal pathway [hypothalamic signal transduction-activated transcript (Stat3)] and results in increased intake of oxygen and increased expenditure of energy. Additionally, it was proven that vaspin's activity is significant as it acts as serine protease inhibitor because using vaspin with mutation in serpin domain partially neutralizes this effect [37].

In physiological conditions vaspin is characterized by pulsatile secretion and its level is approximately twice as high among women compared to men [38]. Concentration of this adipocytokine in blood after overnight fasting is low, but 1-2 hours before expected meal it starts to rise [39]. There are no studies on possible disorders in vaspin secretion in anorexia nervosa during various times of the day or modifications correlated with food consumption. The presented study, as well as the few analyses available in the literature on the subject, involved overnight fasting [16]. Moreover, it cannot be ruled out that patients with anorexia nervosa may have vaspin resistance, a mechanism that is similar to ghrelin resistance, which was confirmed for this disease; increased levels of ghrelin in persons with anorexia are most probably a compensating defensive mechanism which, nevertheless, does not result in the expected increase of food intake [40–42]. In the light of the findings from in vitro and in vivo experiments it could be thus suggested that our results connected with concomitant changes vaspin levels in patients with small BMI parameters, albeit not direct, indicate rather the involvement of this protein in weight loss in this disorders (but and/or vice versa mechanisms are not excluded).

In comparison to the control group, antioxidant status in the examined patients with extreme anorexia nervosa is low and indicates predominance of prooxidative processes over antioxidative processes. Results of studies on this issue are inconclusive, although in majority of the cases they show similar correlations as described in this study. Meta-analysis carried out in 2015 showed that patients suffering from this disease have a high concentration of oxidative stress marker in blood, namely, apolipoprotein B, with comparable levels of antioxidative protein, albumin, in blood if compared to the results of persons with normal body weight. Some studies also reveal lower superoxide dismutase activity in blood and one analysis revealed increased catalase activity and high level of Thiobarbituric Acid Reactive Substances (which indicates elevated lipid peroxidation) as well as low concentrations of glutathione and free cysteine in blood [43]. Other studies have shown high production of reactive oxygen species in anorexia nervosa [44].

Results of meta-analysis based on 6 studies carried out on female patients with AN show interrelation between antioxidant status and nutritional status. After 8 weeks of oral nutritional therapy concentration, levels of apolipoprotein B (one of the prooxidative parameters) decreased, whereas albumin concentration levels in blood increased (with confirmed antioxidative effect). One of the studies (included in the mentioned meta-analysis) also demonstrated that the increase of total antioxidants level after nutritional treatment, which was applied, could be linked to increased catalase activity [45, 46]. The analyses have shown that increased activity of this enzyme was noted in the case of patients with AN for whom recovery was rather fast (less than 3 months) versus those patients who required longer treatment [45]. The complexity of oxidative and reductive processes that take place before and during nutritional treatment in anorexia nervosa needs to be emphasized. With increased catalase activity another antioxidant enzyme proved to be less active, namely, superoxide dismutase, which may indicate that prooxidative processes are still very active and/or that defensive mechanisms of patients with anorexia are gradually weakening even after body weight was partially stabilized (average increase of BMI by 2 kg/m^2^) [46]. The parameter that we applied in our studies, total antioxidant status, is a parameter that takes into account the dynamics of changes in antioxidant capacity of blood serum/plasma or other body fluids and also involves antioxidative enzymes, as well as low molecular enzymes; its assessment helps describe antioxidative characteristics of complex biological systems more effectively than a sum of concentrations/activity of all antioxidants measured individually [47].

Adjusted serum neuropeptide B level according to Body Mass compared to parallel adjusted serum vaspin and TAS levels proved to be a significant predictor for extreme anorexia nervosa (according to ROC curves). Since most of the recognized diagnostic markers in medicine are characterized by AUC value according to ROC curve between 0.8 and 0.9 and with values that are 0.7 and above the predictors are deemed satisfactory, adjusted serum NPB level may be considered to be a good marker. Lower sensitivity and specificity were noted in the case of adjusted serum VAS level, whereas adjusted serum TAS level does not seem to be useful biomarker at this stage of the disease [48]. On the basis of analyses carried out with regard to anorexia nervosa and other disease units it may be assumed that vaspin and total antioxidant status parameters juxtaposed with Body Mass Index depend on a number of factors, including inflammatory condition, carbohydrate metabolism, or hormonal disorders; hence one should be cautious when interpreting them [16, 49–51].

## 5. Method Limitations

When designing the study a minimum number of assays were determined (taking into account calculations from statistical software) on the basis of pilot studies results and with the assumption that the test's strength is 0.9 and type I error is 0.05 (parameters often used in medical studies). Having that in mind, the study involved 46 patients with extreme anorexia nervosa; nevertheless, it is worth considering expanding the analyses by including larger study cohorts to confirm research results, especially as there exists a significant variability of study results. This is no easy task in the case of patients with anorexia nervosa at the extreme stage of the disease, especially in the paediatric population (when the clinical condition often makes it impossible to even collect a blood sample). There were some limitations concerning anthropometric analyses in the case of adolescents. The latter involved taking body weight and height measurements to define anthropometric ratios (Body Mass Index z-score and Body Mass Index-for-age percentile), which is recommended to assess nutritional status but without the use of electrical bioimpedance, as it is not recommended to persons under 18 years of age [52].

## 6. Conclusions

The heterogeneity of determinants in eating habits in AN indicates that the nature of the disturbance is a complex one. In detailed diagnostics of anorexia nervosa (especially in the cases of the extreme form of this disorder) it is worth considering testing neuropeptide B and vaspin levels in serum.

## Figures and Tables

**Table 1:** Anthropometric and biochemical analyses in anorexia nervosa group (AN) and control group (CONTR).

Parameter [unit]	AN (n=46)	CONTR (n=30)	P value
Age [years]	15.7±1.8 16.0 (14.0;17.0)	15.4±1.5 15.0 (14.0;17.0)	NS

Body Mass [kg]	36.6±4.5 36.0 (33,0;40.0)	56.0±9.9 54.0 (49,6;58.0)	<0.000001

High [m]	1.616±0.067 1.620 (1.560;1.670)	1.650±0.042 1.640 (1.620;1.680)	NS

BMI [kg/m^2^]	13.97±1.14 14.20 (13.05;14.70)	20.54±3.57 20.58 (17.91;21.86)	<0.000001

BMI z-score	-4.019±1.501 -3.900 (-4.690;-2.890)	-0.129±1.024 -0.240 (-0.790;0.450)	<0.000001

Body Mass Index - for - age percentile	0.45±0.92 0.10 (0.10;0.10)	46.07±29.65 41.00 (21.00;67.00)	<0.000001

Neuropeptide B [ng/mL]	0.448±0.327 0.258 (0.135;0.885)	0.278±0.248 0.159 (0.110;0.295)	<0.05

Neuropeptide W [ng/mL]	0.273±0.145 0.196 (0.147;0.313)	0.203±0.120 0.161 (0.147;0.221)	NS

Parameters were presented as means±standard deviations and medians (with upper and lower quartile). n: number of people; p: level of statistical significance in Mann–Whitney test or Welch test (for, respectively, parametric and nonparametric date distributions); BMI: Body Mass Index; BMI z-score: Body Mass Index z-score; NS: not statistically significant difference.

**Table 2:** Characteristics of ROC curves for adjusted serum neuropeptide B, vaspin, and Total Antioxidant Status levels according Body Mass Index for pairs of studied groups (AN; n=46 versus CONTR; n=30).

Parameter [unit]	Sensitivity	Specificity	Cut-off value	AUC	SD (AUC)	95% CI	P
Adjusted neuropeptide B [ng/mL/kg/m^2^]	91%	37%	14.67	0.700	0.060	0.581-0.819	<0.001

Adjusted vaspin [ng/mL/kg/m^2^]	87%	50%	0.02	0.647	0.067	0.516-0.777	<0.003

Adjusted Total Antioxidant Status [mmol/L/kg/m^2^]	52%	75%	0.06	0.583	0.065	0.456-0.711	NS

AN: group with anorexia nervosa; CONTR: control group; n: number of persons; cut-off value: point on ROC (Receiver Operating Characteristic) curve; AUC: Area Under Curve of Receiver Operating Characteristic curve; SD (AUC): standard deviation of AUC; p: level of statistical significance; NS: not statistically significant difference.

## References

[1] Kostro K., Lerman J. B., Attia E. (2014). The current status of suicide and self-injury in eating disorders: a narrative review. *Journal of Eating Disorders*.

[2] Keski-Rahkonen A., Mustelin L. (2016). Epidemiology of eating disorders in Europe: Prevalence, incidence, comorbidity, course, consequences, and risk factors. *Current Opinion in Psychiatry*.

[3] Lian Q., Zuo X., Mao Y. (2017). Anorexia nervosa, depression and suicidal thoughts among Chinese adolescents: a national school-based cross-sectional study. *Environmental Health and Preventive Medicine*.

[4] Coniglio K. A., Becker K. R., Franko D. L. (2017). Won't stop or can't stop? Food restriction as a habitual behavior among individuals with anorexia nervosa or atypical anorexia nervosa. *Eating Behaviors*.

[5] Nagata J. M., Golden N. H., Peebles R. (2017). Assessment of sex differences in body composition among adolescents with anorexia nervosa. *Journal of Adolescent Health*.

[6] American Psychiatric Association (2013). *Diagnostic and Statistical Manual of Mental Disorders*.

[7] Treasure J., Cardi V. (2017). Anorexia nervosa, theory and treatment: where are we 35 years on from hilde bruch's foundation lecture?. *European Eating Disorders Review*.

[8] Grzelak T., Dutkiewicz A., Paszynska E., Dmitrzak-Weglarz M., Slopien A., Tyszkiewicz-Nwafor M. (2017). Neurobiochemical and psychological factors influencing the eating behaviors and attitudes in anorexia nervosa. *Journal of Physiology and Biochemistry*.

[9] De Filippo E., Marra M., Alfinito F. (2016). Hematological complications in anorexia nervosa. *European Journal of Clinical Nutrition*.

[10] DeBoer M. D. (2011). What can anorexia nervosa teach us about appetite regulation?. *Nutrition Journal *.

[11] Tanaka H., Yoshida T., Miyamoto N. (2003). Characterization of a family of endogenous neuropeptide ligands for the G protein-coupled receptors GPR7 and GPR8. *Proceedings of the National Acadamy of Sciences of the United States of America*.

[12] Kelly M. A., Beuckmann C. T., Williams S. C. (2005). Neuropeptide B-deficient mice demonstrate hyperalgesia in response to inflammatory pain. *Proceedings of the National Acadamy of Sciences of the United States of America*.

[13] Mondal M. S., Yamaguchi H., Date Y. (2003). A role for neuropeptide W in the regulation of feeding behavior. *Endocrinology*.

[14] Date Y., Mondal M. S., Kageyama H. (2010). Neuropeptide W: an anorectic peptide regulated by leptin and metabolic state. *Endocrinology*.

[15] Diaz R. (2010). An anorectic role for NPW?. *Nature Reviews Endocrinology*.

[16] Oswiêcimska J., Suwaa A., Swiêtochowska E. (2016). Serum vaspin concentrations in girls with anorexia nervosa. *Journal of Pediatric Endocrinology and Metabolism*.

[17] Ostrowska Z., Ziora K., Oswiecimska J., Oświęcimska J. (2016). Vaspin and selected indices of bone status in girls with anorexia nervosa. *Endokrynologia Polska*.

[18] Paszynska E., Tyszkiewicz-Nwafor M., Slopien A., Dmitrzak-Weglarz M., Dutkiewicz A., Grzelak T. (2017). Study of salivary and serum vaspin and total antioxidants in anorexia nervosa. *Clinical Oral Investigations*.

[19] Li K., Li L., Yang M. (2011). Short-term continuous subcutaneous insulin infusion decreases the plasma vaspin levels in patients with type 2 diabetes mellitus concomitant with improvement in insulin sensitivity. *European Journal of Endocrinology*.

[20] Heiker J. T. (2014). Vaspin (serpinA12) in obesity, insulin resistance, and inflammation. *Journal of Peptide Science*.

[21] The ICD-10 classification of mental and behavioral disorders diagnostic criteria for research. http://www.who.int/classifications/icd/en/.

[22] Palczewska I., Niedzwiedzka Z. (2001). Somatic development indices in children and youth of Warsaw. *Medycyna Wieku Rozwojowego*.

[23] Davies J. E., Cockfield A., Brown A., Corr J., Smith D., Munro C. (2017). The medical risks of severe anorexia nervosa during initial re-feeding and medical stabilisation. *Clinical Nutrition ESPEN*.

[24] White H. J., Haycraft E., Madden S. (2017). Parental strategies used in the family meal session of family-based treatment for adolescent anorexia nervosa: Links with treatment outcomes. *International Journal of Eating Disorders*.

[25] Manual Protocol for human vaspin. http://www.ibl-america.com/pdf/elisa/E106.pdf.

[26] Species-Cross-Reactions for Isolated Products of Mediagnost. http://mediagnost.de/wordpress/wp-content/uploads/2017/04/Mediagnost-Species-Cross-Reactions-090913.pdf.

[27] Manual Protocol for human NPB and human NPW. http://www.srbooo.com.

[28] Samson W. K., Baker J. R., Samson C. K., Samson H. W., Taylor M. M. (2004). Central neuropeptide B administration activates stress hormone secretion and stimulates feeding in male rats. *Journal of Neuroendocrinology*.

[29] Skrzypski M., Pruszyńska-Oszmałek E., Ruciński M. (2012). Neuropeptide B and W regulate leptin and resistin secretion, and stimulate lipolysis in isolated rat adipocytes. *Regulatory Peptides*.

[30] Ishii M., Fei H., Friedman J. M. (2003). Targeted disruption of GPR7, the endogenous receptor for neuropeptides B and W, leads to metabolic defects and adult-onset obesity. *Proceedings of the National Acadamy of Sciences of the United States of America*.

[31] Loomba-Albrecht L. A., Styne D. M. (2009). Effect of puberty on body composition. *Current Opinion in Endocrinology, Diabetes and Obesity*.

[32] Fujii R., Yoshida H., Fukusumi S. (2002). Identification of a neuropeptide modified with bromine as an endogenous ligand for GPR7. *The Journal of Biological Chemistry*.

[33] Shimomura Y., Harada M., Goto M. (2002). Identification of neuropeptide W as the endogenous ligand for orphan G-protein-coupled receptors GPR7 and GPR8. *The Journal of Biological Chemistry*.

[34] Li H., Kentish S. J., Kritas S. (2013). Modulation of murine gastric vagal afferent mechanosensitivity by neuropeptide W. *Acta Physiologica*.

[35] Li H., Feinle-Bisset C., Frisby C., Kentish S., Wittert G. A., Page A. J. (2014). Gastric neuropeptide W is regulated by meal-related nutrients. *Peptides*.

[36] Herpertz-Dahlmann B., Seitz J., Baines J. (2017). Food matters: how the microbiome and gut–brain interaction might impact the development and course of anorexia nervosa. *European Child & Adolescent Psychiatry*.

[37] Kim M.-S., Youn B.-S. Method of preventing or treating body weight-related disorders by employing vaspin. http://www.google.com/patents/WO2011138977A1?cl=en.

[38] Koiou E., Kalaitzakis E., Tziomalos K. (2011). Vaspin: a novel adipokine, member of the family of serine protease inhibitors. *Aristotle University Medical Journal*.

[39] Jeong E., Youn B.-S., Kim D. W. (2010). Circadian rhythm of serum vaspin in healthy male volunteers: relation to meals. *The Journal of Clinical Endocrinology & Metabolism*.

[40] Nedvídková J., Krykorková I., Barták V. (2003). Loss of meal-induced decrease in plasma ghrelin levels in patients with anorexia nervosa. *The Journal of Clinical Endocrinology & Metabolism*.

[41] Tolle V., Kadem M., Bluet-Pajot M.-T. (2003). Balance in Ghrelin and leptin plasma levels in anorexia nervosa patients and constitutionally thin women. *The Journal of Clinical Endocrinology & Metabolism*.

[42] Cruz-Domínguez M. P., Cortés D. H. M., Zarate A. (2014). Relationship of ghrelin, acid uric and proinflammatory adipocytokines in different degrees of obesity or diabetes. *International Journal of Clinical and Experimental Medicine*.

[43] Solmi M., Veronese N., Manzato E. (2015). Oxidative stress and antioxidant levels in patients with anorexia nervosa: a systematic review and exploratory meta-analysis. *International Journal of Eating Disorders*.

[44] Victor V. M., Rovira-Llopis S., Saiz-Alarcon V. (2014). Altered mitochondrial function and oxidative stress in leukocytes of anorexia nervosa patients. *PLoS ONE*.

[45] Oliveraslópez M.-J., Ruiz-Prieto I., Bolaños-Ríos P., De la Cerda F., Martín F., Jáuregui-Lobera I. (2015). Antioxidant activity and nutritional status in anorexia nervosa: effects of weight recovery. *Nutrients*.

[46] Solmi M., Veronese N., Luchini C. (2016). Oxidative stress and antioxidant levels in patients with anorexia nervosa after oral re-alimentation: a systematic review and exploratory meta-analysis. *Aristotle University Medical Journal*.

[47] Li Y., Browne R. W., Bonner M. R., Deng F., Tian L., Mu L. (2014). Positive relationship between total antioxidant status and chemokines observed in adults. *Oxidative Medicine and Cellular Longevity*.

[48] Mezaour A. D., Klopotek M., Wierzchon S. T., Trojanowski K. Filtering Web documents for a thematic warehouse case study: aDots a food risk date warehouse (extended). https://www.springer.com/gp/book/9783540250562.

[49] Körner A., Neef M., Friebe D. (2005). Vaspin is related to gender, puberty and deteriorating insulin sensitivity in children. *International Journal of Obesity*.

[50] Solmi M., Veronese N., Favaro A. (2015). Inflammatory cytokines and anorexia nervosa: a meta-analysis of cross-sectional and longitudinal studies. *Psychoneuroendocrinology*.

[51] Sperling M., Grzelak T., Pelczynska M. (2016). Concentrations of omentin and vaspin versus insulin resistance in obese individuals. *Biomedicine & Pharmacotherapy*.

[52] Luque V., Escribano J., Zaragoza-Jordana M. (2014). Bioimpedance in 7-year-old children: validation by dual x-ray absorptiometry—part 2: assessment of segmental composition. *Annals of Nutrition and Metabolism*.

